# Why Solvent Response Contributions to Solvation Free Energies Are Compatible with Ben-Naim’s Theorem

**DOI:** 10.1021/acs.jctc.3c00655

**Published:** 2023-11-10

**Authors:** Leonard P. Heinz, Helmut Grubmüller

**Affiliations:** Department of Theoretical and Computational Biophysics, Max-Planck Institute for Multidisciplinary Sciences, 37077 Göttingen, Germany

## Abstract

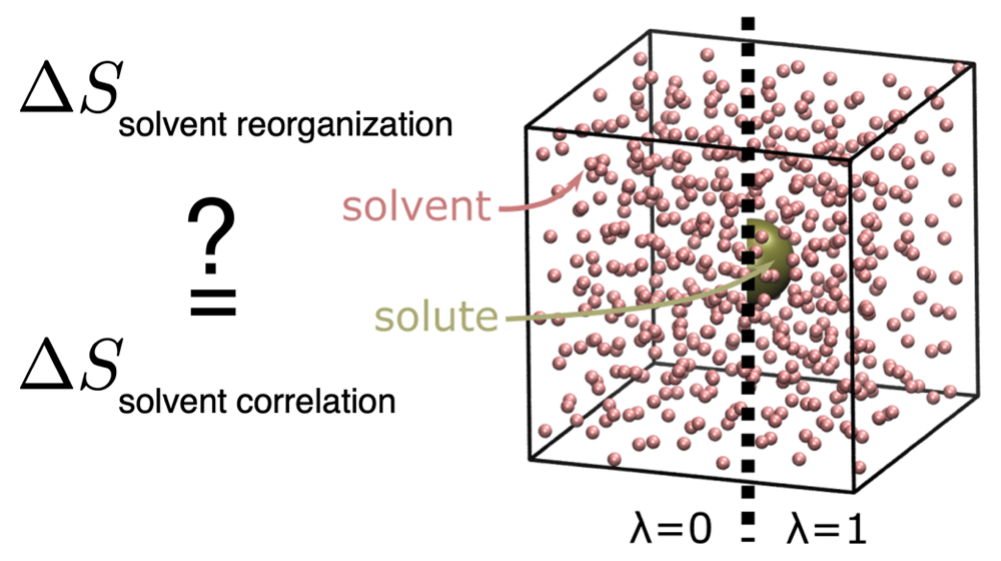

We resolve a seeming paradox arising from a common misinterpretation of Ben-Naim’s theorem, which rests on the decomposition of the Hamiltonian of a molecular solute/solvent system into solute–solvent and solvent–solvent interactions. According to this theorem, the solvation entropy can also be decomposed into a solute–solvent term and a remaining solvent–solvent term that is commonly referred to as the solvent reorganization term. Crucially, the latter equals the average solvent–solvent interaction energy such that these two solvent–solvent terms cancel and thus do not change the total solvation free energy. This analytical result implies that changes in the solvent–solvent interactions cannot contribute to any thermodynamic driving force. The solvent reorganization term is often identified with the contribution of many-body solvent correlations to the solvation entropy, which seems to imply that these correlations, too, cannot contribute to solvation. However, recent calculations based on atomistic simulations of a solvated globular protein and spatially resolved mutual information expansions revealed substantial contributions of many-body solvent correlations to the solvation free energy, which are not canceled by the enthalpy change of the solvent. Here, we resolved this seeming contradiction and illustrate by two examples—a simple Ising model and a solvated Lennard-Jones particle—that the solvent reorganization entropy and the actual entropy contribution arising from many-body solvent correlations differ both conceptually and numerically. Whereas the solvent reorganization entropy in fact arises from both solvent–solvent as well as solute–solvent interactions and thus has no straightforward intuitive interpretation, the mutual information expansion permits an interpretation in terms of the entropy contribution of solvent–solvent correlations to the solvation free energy.

## 1 Introduction

The hydrophobic effect is an essential driving force for many processes in nature, such as phase separation, membrane formation,^1−3^ or the function and folding of proteins.^4,5^ Despite its significance, the hydrophobic effect is not yet fully understood from first-principles, and hence, its molecular explanation remains controversial.^6^ The early “iceberg” hypothesis by Frank and Evans,^7^ for example, turned out to be equally popular and controversial. Frank and Evans explained the unfavorable solvation free energy of hydrophobic solutes in water by an entropic penalty due to an ordered “iceberg” structure of water molecules that forms around the solute. The term “iceberg” is not meant to be taken literally, but rather refers to a higher ordering of the first few solvation shells compared to bulk water.^7,8^

Indeed, the hydrophobic effect has been shown to be mainly entropy-driven^4^ and such ordered structures have been found around hydrophobic solutes.^8−11^ However, in seminal papers, Ben-Naim^12−14^ has analytically proven that the so-called solvation reorganization term or cavity formation term occurs in both the enthalpic and the entropic part of the solvation free energy; as a result, this term cancels out in the solvation free energy and, therefore, structural changes to the solvent that give rise to changes in solvent–solvent interactions cannot be a thermodynamic driving force of solvation. Subsequently, Yu and Karplus^15^ have obtained the same result via a different route for the special case of pairwise additive interactions.

This important theorem has led to the general understanding that upon solvation, the enthalpy change arising from solvent–solvent interactions and the entropy change due to water ordering cancel as well and thus, too, cannot contribute to the net free energy change of solvation. As we will discuss below, this conclusion rests on the wrong interpretation of the solvent reorganization term as an entropy change of the solvent or, more specifically, entropy changes due to many-body solvent correlations. Indeed, ordered structures of water molecules around hydrophobic solutes are still implied as the cause of hydrophobicity,^16,17^ a notion that has then been criticized by others.^14,18^ Overall, this seeming contradiction caused considerable confusion and still does.

We have recently addressed this issue from a simulation perspective and have calculated enthalpic and entropic contributions to the folding free energy of crambin.^19^ Specifically, we calculated and compared the solvation shell enthalpies and entropies of the solvated folded conformation of crambin and a molten-globule-like conformation of this prototypic globular protein. To directly quantify many-body solvent correlations, we used the method Per|Mut,^20,21^ which employs a mutual information expansion (MIE)^22−25^
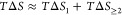
1into single-molecule entropies Δ*S*_1_ and entropy contributions Δ*S*_≥2_ arising from correlations between pairs and triples of—mostly nearby—solvent molecules. This entropy decomposition differs from the one put forward by Ben-Naim and Yu and Karplus and offers a more direct and microscopically intuitive interpretation. For example, the single-molecule entropy Δ*S*_1_ contribution to the solvation free energy in many cases reflects direct interactions between the solvent and the solute, whereas the entropy contributions due to solvent–solvent correlations provide direct microscopic insights into how the solvent structure and fluctuations are affected and, in turn, contribute to the solvation free energy. Hence, these calculations also offer the chance to test whether entropy changes due to many-body solvent correlations are canceled by changes in solvent–solvent interactions.

In our molecular dynamics (MD) simulations,^19^ the molten-globule-like conformation of crambin showed many hydrophobic residues, which are buried within the folded conformation, exposed to the solvent. Relative to the native fold, we observed indeed a marked entropic free energy contribution to the solvation free energy due to strongly correlated water molecules in the innermost solvation shells.

Here, we show that this finding is, in fact, perfectly compatible with the Ben-Naim theorem. Our analysis will, further, provide a deeper understanding of the contribution of the solvent response to the solvation free energy and also clarify the notion that structural changes of the solvent cannot affect the solvation free energy.^12^ We will illustrate our reasoning by a simple Ising model example that can be exhaustively enumerated as well as by a more realistic example of a Lennard-Jones particle solvated in liquid argon.

## 2 Theory

### 2.1 Canonical Decomposition

Ben-Naim^12−14^ has proven that the change of average solvent–solvent interaction energies upon solvation is exactly compensated by a corresponding entropy term, such that there is no net free energy contribution. Later, Yu and Karplus^15^ obtained essentially the same result for the less general case of pairwise additive solute–solvent and solvent–solvent interactions by considering a solvation process described by the coupling parameter λ (λ = 0: not solvated, λ = 1: fully solvated).

In particular, they demonstrated that for a Hamiltonian
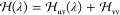
2consisting of pairwise solute–solvent (uv) and solvent–solvent (vv) interactions, the internal energy (Δ*U*) and entropy (Δ*S*) changes can be expressed as
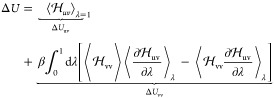
3a
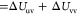
3b
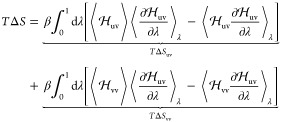
4a
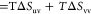
4bWhereas the internal energy and entropy parts Δ*U*_uv_ and *T*Δ*S*_uv_ only contain solute–solvent interactions , the remaining terms are referred to as solvent–solvent terms, Δ*U*_vv_ and *T*Δ*S*_vv_, respectively. The important finding by Ben-Naim and Yu and Karplus is that these two terms, also referred to as or solvent reorganization terms, are identical and thus cancel in the net free energy difference
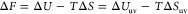
5which thus only contains the so-called solute–solvent terms.

Note, however, that whereas the interpretation of Δ*U*_vv_ as the change of the average solvent–solvent interaction is straightforward, there is no similarly intuitive microscopic interpretation for the (canceling) entropy term *T*Δ*S*_vv_. Although it arises canonically as the second term in eq 4a, and despite the common subscript “solvent–solvent”, Δ*S*_vv_ does not describe the entropy change of the solvent due to its changed ordering. In fact, the separation into solute–solvent and solvent–solvent interactions does not imply a unique additive separation of corresponding entropy contributions. We think that this misconception is the root of long-standing and widespread confusion.

In particular, the seeming absence of solvent–solvent terms in the free-energy balance has led to the widely held belief that the solvent response to the presence of a solute, e.g., solvent rearrangements such as the Frank and Evans “icebergs”, cannot contribute as a thermodynamic driving force.^14,26^

### 2.2 Mutual Information Expansion

Additionally, although the two terms Δ*S*_uv_ and Δ*S*_vv_ are of course well-defined, it is misleading to interpret these entropy terms, which are inherently ensemble properties, as representing the entropy contribution arising separately from solute–solvent and solvent–solvent interactions, respectively, as defined in eq 2. For example, the term Δ*S*_vv_ in eq 4a contains solute–solvent interactions  not only implicitly via ensemble averages but also explicitly.

As an alternative, and to gain physical insights into the solvation process that can be interpreted in a more straightforward manner, we suggest to use a MIE,^22−25^ as, e.g., implemented in the recently developed method Per|Mut.^20,21^

Accordingly, the total solvent entropy is decomposed into single-body entropies, akin to an ideal-gas term, and multibody correlations
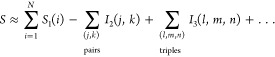
6a
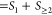
6bwhere *S*_1_(*i*) are the single-body entropies of the (three-dimensional) probability distributions of molecules 1, ..., *N* and *I*_2_(*j*, *k*) and *I*_3_(*l*, *m*, *n*) are the two-body and three-body mutual information terms of molecule pairs and triples, respectively. These terms are defined as
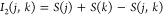
7a
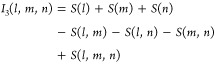
7band represent the entropy change due to two- and three-body correlations, respectively. In this notation, *S*(*j*, *k*) and *S*(*l*, *m*, *n*) are the entropies of the (six- and nine-dimensional) marginal distributions of the full configuration space density ϱ with respect to molecule pairs *j*, *k* and triples *l*, *m*, *n*, respectively, e.g.
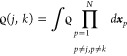
8

A full MIE up to the *N*-body correlation term yields an exact entropy decomposition. In our numerical approach below, evaluation of the respective integrals would require sampling over the full 3*N*-dimensional configuration space, which is impractical. We therefore truncated the expansion after the three-body correlations to obtain a good approximation of entropy, neglecting higher-order terms. For short-ranged interactions, these have indeed been demonstrated to be small.^27^

## 3 Methods

### 3.1 Ising Model

**Figure 1:**
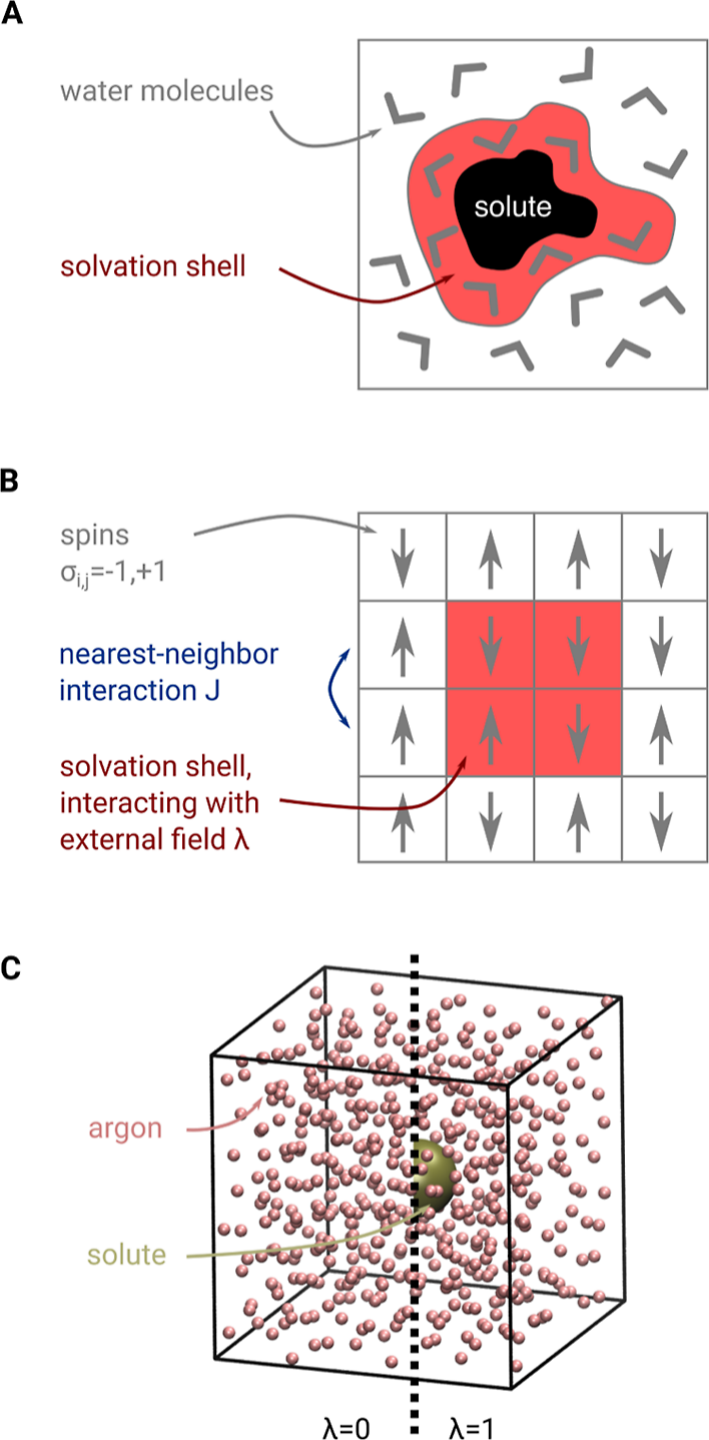
(A) Sketch of a solute (black) in water (gray angles), where the solvation shell (red area) interacts directly with the protein. (B) Sketch of a 4 × 4 Ising model, serving as a simplified model of solute–solvent/solvent–solvent interactions. Here, spins (gray arrows) represent water molecules. The four spins shaded in red interact with the solute; this interaction is described by an external field λ that acts on the four spins. (C) As a more realistic model system, argon-type atoms (red) are coupled to a solute Lennard-Jones sphere (yellow). On the left (λ = 0), the solute is decoupled from the argon solvent; on the right (λ = 1), the solute is fully solvated.

To assess the solvent response to a solute (e.g., a protein), as sketched in Figure 1A, we first considered the simple 4 × 4 subcritical Ising model sketched in Figure 1B. In this model, each spin σ_*i*,*j*_ = −1, +1 interacts with its nearest neighbors with an interaction strength *J* = 0.2 under periodic boundary conditions. Here, the spins mimic a solvent, with the four most center spins (shaded in red) interacting with an external field λ, which mimics the interaction with a solute. Note that because the solute is described purely by these interactions, the Ising model depicted in Figure 1B does not contain any explicit solute degrees of freedom.

Accordingly, the Hamiltonian reads
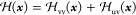
9a
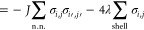
9bwhere the first sum runs over all nearest neighbors, and the second sum runs over the spins shaded in red (i.e., the “solvation shell”, the solute is not shown in Figure 1B). The probability of each state ***x*** ∈ ***X*** = [−1, +1]^4×4^ reads
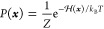
10with the partition function *Z* chosen such that ∑_***x***∈***X***_*P*(***x***) = 1.

The entropy *S* and the average solvent–solvent interaction energy therefore read
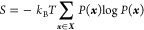
11
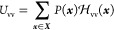
12

Following Yu and Karplus,^15^ all solute–solvent and solvent–solvent entropy changes were calculated according to eq 4a. These values are therefore subject to a small integration error due to the required numerical integration, for which we used 251 discrete λ-intermediates. For the Ising model, all calculations were carried out with unitless energies, i.e., *k*_B_ = *T* = β = 1.

### 3.2 Argon

#### 3.2.1 MD Simulations

To quantify the response of an argon-type liquid to a Lennard-Jones solute, two systems were simulated: an unsolvated system containing 512 argon-type atoms and a solvated system with an additional immobilized van der Waals sphere as a “solute”. All MD simulations were carried out using the software package Gromacs 2020.6^28−32^ with a leapfrog integrator with a 2 fs time step. The van der Waals parameters of argon were taken from the CHARMM36m force field.^33−35^ The Lennard-Jones^36^ parameters (particle size σ and potential depth ϵ) for the model solute were chosen as twice as those of argon to enhance the statistical significance of average energy differences. All van der Waals interactions were switched between 1.0 and 1.2 nm, and no dispersion correction was applied. To immobilize the solute at the center of the simulation box, freeze-options within Gromacs were used. During all simulation runs, the temperature was kept at 120 K using the V-rescale thermostat^37^ with a time constant of 0.1 ps.

The unsolvated (pure argon) system was equilibrated at 1 bar pressure in a 20 ns *NPT*-run using the Berendsen barostat,^38^ resulting in a (3.073 nm)^3^ cubic simulation box. The second, solvated system was prepared by adding the solute to the simulation box and allowing for a further equilibration, lasting 10 ns under *NVT* conditions. For both systems, production runs, each lasting 4 μs, were carried out under *NVT* conditions. For subsequent analysis, configurations were stored every 10 ps, resulting in trajectories consisting of 4 × 10^5^ frames each.

#### 3.2.2 Entropy Calculation

Entropy contributions were calculated using the method Per|Mut,^20,21^ which utilizes a permutation reduction^39,40^ and a MIE^22−25^ into one-, two-, and three-body correlations. For permutation reduction, 50 different simulation snapshots were randomly selected as reference structures, and a MIE was carried out using each of the permutationally reduced trajectories. In the MIE, the mutual information between all pairs of argon atoms was taken into account; triple-wise mutual information terms were cut off at an average distance of 0.5 nm after permutation reduction.^20,21^ All MIE orders were calculated using a k-nearest-neighbor algorithm with a value of *k* = 1.

From the resulting entropy difference Δ*S*_MIE_ between the unsolvated system and the solvated system, the free energy difference Δ*F*_MIE_ = Δ*U* – *T*Δ*S*_MIE_ was calculated, where the internal energy difference Δ*U* was obtained directly from the average interaction energies in the simulation runs.

Solute–solvent and solvent–solvent entropy differences Δ*S*_uv_ and Δ*S*_vv_, respectively, were calculated by thermodynamic integration (TI) from the unsolvated state to the solvated state using 200 equidistant windows, each lasting 200 ns. As a control for the Per|Mut results, the total entropy difference Δ*S*_TI_ = Δ*S*_uv_ + Δ*S*_vv_ and the free energy change
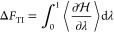
13were calculated using standard TI.

Errors of the internal energies were calculated as , where σ_U_ are the standard deviations of the respective interaction energies from *N*_f_ = 400 × 10^3^ simulation frames. Due to the long interval of 10 ps between frames, these were considered statistically independent. Similarly, Per|Mut errors were estimated as the standard errors resulting from the 50 permutationally reduced simulation trajectories. TI errors were estimated from the difference between two independent sets of TI simulation runs with identical input parameters but different initial (random) velocities but turned out to be negligible for all further analyses.

## 4 Results and Discussion

To investigate the seeming contradiction between the Ben-Naim theorem and the free energy effects of increased solvent correlations observed for the crambin^19^ solvent shell, we calculated the relevant contributions of the solvent response to the solvation free energy for two simple model systems, for which sampling errors are either absent or can be neglected. Specifically, we will compare all free energy contributions of the Yu et al. decomposition (eqs 3a–5) with the MIE (eq 1).

We will first consider an idealized solvation process for an Ising model, for which all relevant quantities can be exhaustively enumerated, such that the results are exact to numerical precision. Subsequently, we will consider a liquid argon-type Lennard-Jones system with a van der Waals solute, the relaxation times of which are short with respect to simulation times, such that for this, more realistic system sampling errors can be assumed to be very small with respect to the relevant energy and entropy differences.

### 4.1 Ising Model

As a simple illustrative model of a solute in a solvent (Figure 1A), we consider the 4 × 4 subcritical Ising model shown in Figure 1B. Here, each spin represents a solvent molecule that interacts with its nearest neighbors. The effects of a solute are modeled by an external field with strength λ that acts on the “solvation shell” (red), consisting of the four spins at the center.

**Figure 2:**
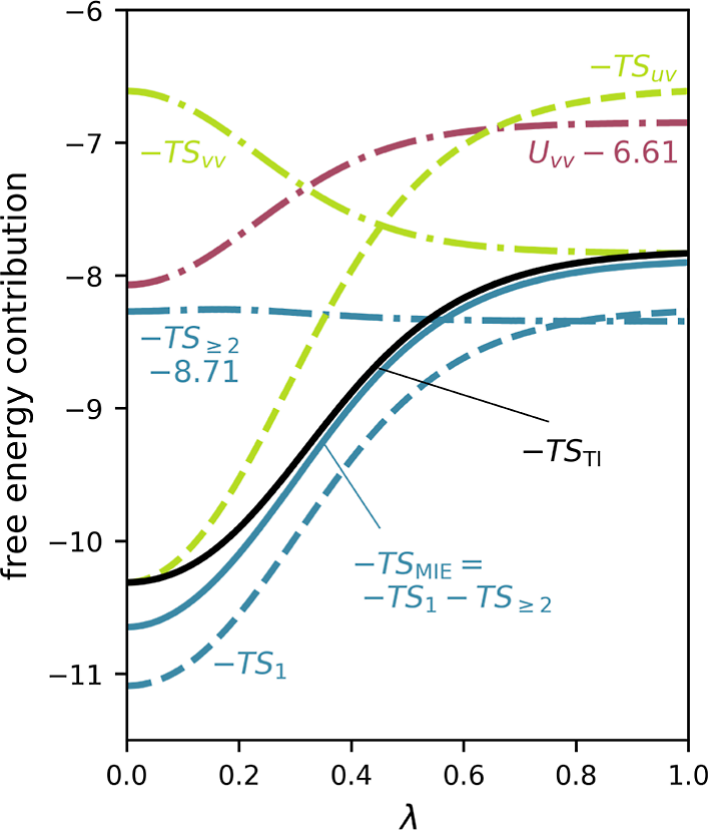
Thermodynamic quantities of the Ising model as a function of the external field λ. Individual entropic (green and blue) and enthalpic (red) contributions are shown as dashed or dashed-dotted lines, and the respective sums and total entropies are shown as solid lines. For reference, the precise entropy contribution (−*TS*_TI_) is shown as a solid black line. Entropies obtained via Per|Mut MIE are colored blue; entropies representing the Ben-Naim decomposition are shown in green. Labels mark the individual contributions discussed in the text. For a better visual representation, *U*_vv_ and −*TS*_≥2_ are shifted by 6.61 and 8.71 units, respectively.

Figure 2 shows the exact relevant thermodynamic quantities as a function of the coupling parameter λ, calculated by full enumeration as described in Section 3.1. As expected, with the increase in coupling to the solvent, the total entropic free energy contribution −*TS*_TI_ (black line) becomes less favorable (i.e., it increases) and eventually saturates at an entropy difference of −*T*Δ*S*_TI_ = 2.48 between fully solvated (λ = 1) and fully decoupled (λ = 0). This contribution is dominated by the unfavorable solute–solvent contribution −*T*Δ*S*_uv_ = 3.70 (green dashed line), which is partially compensated by the favorable solvent–solvent contribution −*T*Δ*S*_vv_ = −1.22 (green dashed-dotted line).

As shown by the dashed-dotted red line in Figure 2, the average solvent–solvent interaction energies *U*_vv_ increase for increasing coupling parameter λ and thus contribute unfavorably to the free energy change by Δ*U*_vv_ = 1.22. Fully in line with the Ben-Naim theorem (eq 5), *U*_vv_ is indeed precisely compensated by −*TS*_vv_, such that Δ*U*_vv_ – *T*Δ*S*_vv_ = 0 and, hence, Δ*F* = Δ*U*_uv_ – *T*Δ*S*_uv_ (Δ*U*_uv_ = −15.98, not shown in Figure 2).

Does this finding imply that solvent–solvent correlations do not contribute to the solvation free energy? To answer this question, consider the above MIE of entropy, which directly quantifies these correlations. The single-body term −*TS*_1_ (dashed blue line) underestimates the entropy on average by 0.53 units and contributes −*T*Δ*S*_1_ = 2.82 to the overall entropy change. Inclusion of the two- and three-body correlation terms (−*T*Δ*S*_MIE_, solid blue line) improves the approximation markedly, with an average deviation from the exact values below 0.13 units. The correlation terms −*TS*_≥2_ (dashed-dotted light blue line) add a small favorable contribution of −*T*Δ*S*_≥2_ = −0.07 to the overall MIE entropic free energy change (blue solid line) of −*T*Δ*S*_MIE_ = 2.75.

Crucially, the term −*TS*_vv_ differs from the solvent–solvent correlations −*TS*_≥2_ both by definition and, indeed, also numerically, as shown in Figure 2. As a result, −*TS*_uv_ also differs from −*TS*_1_, and the entropy change due to solvent correlations −*T*Δ*S*_≥2_ is not compensated by any canonical internal energy term. This simple example illustrates that, generally, solvent correlations do contribute to the solvation free energy; it also clarifies why this finding is not in conflict with the Ben-Naim theorem.

### 4.2 Argon

Is this subtle but important distinction between −*TS*_vv_ and the actual many-body contribution to the solvation entropy, −*TS*_≥2_, also relevant for more realistic systems? To address this question, we carried out MD simulations of a system comprising 512 argon-type atoms and an immobilized Lennard-Jones “solute”, as described in Section 3.2 (see also Figure 1C). Here, we calculated the free energy change of solvation, as well as the relevant enthalpic and entropic contributions using both Per|Mut and thermodynamic integration.

**Figure 3:**
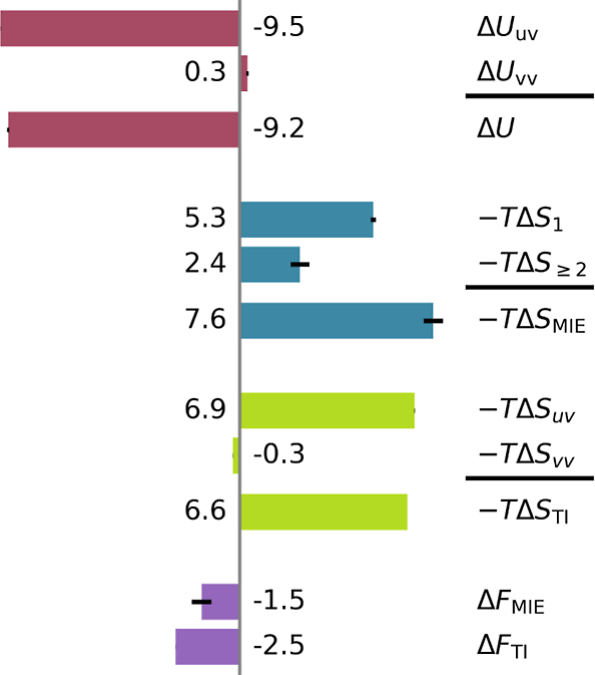
Solvation free energy contributions (in kJ mol^–1^) of the fixed Lennard-Jones solute in an argon-type liquid. Red bars denote the internal energy change and its contributions. Colors correspond to those in Figure 2: blue and green bars denote the entropy change and its contributions, calculated using Per|Mut and TI, respectively. Purple bars show the overall free energy change, as calculated using Per|Mut and TI. Estimated sampling uncertainties are shown as small black bars. Within each colored group, the lowest bar is the sum of the upper 2 bars, as indicated by the horizontal black summation lines.

As shown in Figure 3, the internal energy change Δ*U* upon solvation is favorable and totals −9.2 kJ mol^–1^, to which solvent–solute interactions (Δ*U*_uv_) contribute −9.5 kJ mol^–1^ and solvent–solvent interactions (Δ*U*_vv_) contribute 0.3 kJ mol^–1^. In line with the Ben-Naim theorem, the latter contribution is exactly compensated by −*T*Δ*S*_vv_ = −0.3 kJ mol^–1^, which, also for this system, might suggest that the solvent–solvent interactions and correlations, taken together, do not contribute to the solvation free energy.

However, the many-body entropy contribution −*T*Δ*S*_≥2_ = (2.4 ± 0.4) kJ mol^–1^, calculated using Per|Mut and the MIE, is substantial and contributes a significant fraction to the solvation entropy −*T*Δ*S*_MIE_ = (7.6 ± 0.4) kJ mol^–1^, which is dominated by the reduced volume of the individual argon atoms, −*T*Δ*S*_1_ = (5.3 ± 0.1) kJ mol^–1^.

To test our assumption that four-body and higher correlations not included within −*T*Δ*S*_MIE_ are sufficiently small, we also calculated the relevant entropy terms using TI (green). Indeed, the similar total entropy change of −*T*Δ*S*_TI_ = 6.6 kJ mol^–1^ supports this assumption and shows that the contribution of the higher correlations to the solvation entropy is markedly smaller than the MIE estimate. Also for this more realistic system, the entropy change due to solute–solvent interactions (−*T*Δ*S*_uv_ = 6.9 kJ mol^–1^) dominates, and −*T*Δ*S*_vv_ does not even describe the correct sign of the actual solvent–solvent correlation contribution to the solvation free energy. The remaining difference of ca. 1 kJ mol^–1^ between the MIE and TI solvation entropies is also reflected in the respective total free energies Δ*F*_MIE_ = Δ*U* – *T*Δ*S*_MIE_ = −1.5 kJ mol^–1^ and Δ*F*_TI_ = −2.5 kJ mol^–1^, respectively, underscoring that this difference is mainly due to the truncated MIE expansion rather than sampling uncertainties.

Similar to our findings for the above Ising model, also for the more realistic argon-type system, the two possible entropy decompositions differ significantly. Whereas the small size of the two solvent–solvent terms Δ*U*_vv_ and – *T*Δ*S*_vv_—and in particular their mutual cancellation—seem to show that the solvation of this Lennard-Jones particle is unaffected by the reaction of the solvent, the actual solvent–solvent entropy contributions are substantial and not compensated by any canonical internal energy term.

We conclude that also for the solvation of a Lennard-Jones particle in a Lennard-Jones fluid, the induced solvent reorganization—defined via solvent entropies—contributes markedly to the solvation free energy. We note that the solute has been inserted in the *NVT* ensemble, i.e., with the volume kept constant, which may increase the structural response of the solvent to the insertion of the solute compared to a constant pressure simulation and thus enhance the solvent reorganization terms Δ*U*_vv_ and −*T*Δ*S*_vv_.

## 5 Conclusions

We pointed out that the entropy decomposition by Ben-Naim and Yu et al. into a contribution *S*_uv_ from solute–solvent interactions and a remaining contribution (*S*_vv_) as defined in eq 4a, differs conceptually from direct evaluation—e.g., via a MIE—of entropic solvent–solvent correlation contributions to the solvation free energy. In particular, the term “solvent-reorganization entropy” for Δ*S*_vv_, although canonically defined, may create the wrong impression that any solvent change due to the presence of a solute, however defined, cannot contribute to the net free energy.

Two examples served to illustrate the solution of this seeming paradox. First, a simple semianalytical Ising model, which permitted exhaustive enumeration, establishes that the conceptual difference between Δ*S*_vv_ and Δ*S*_≥2_ actually gives rise to marked numerical differences. Second, our MD simulations of solvation within a Lennard-Jones liquid show that this distinction is also relevant for a more realistic solvation system. For both systems, Δ*S*_vv_ is exactly compensated by the change of average solvent–solvent interactions (Δ*U*_vv_), as required by Ben-Naim’s theorem.

In more general terms, our examples also illustrate an alternative decomposition of entropic contributions to the solvation free energy, which is more accessible to a microscopic interpretation and provides insight into which entropy changes drive or oppose solvation. In particular, the single-molecule entropy difference Δ*S*_1_ describes how restricted spatial or (in the case of, e.g., water solvent) orientational mobility of the solvent molecules due to solute–solvent interactions opposes solvation. In this sense, −*T*Δ*S*_1_ is the natural counterpart of Δ*U*_uv_. In contrast, the higher order terms −*T*Δ*S*_≥2_ = −*T*Δ*S*_2_ – *T*Δ*S*_3_ – ... quantify how (typically) increased translational and orientational solvent–solvent correlations, as, e.g., described by the early “iceberg” hypothesis, reduce the solvent shell entropy and, hence, also oppose solvation.

For the two examples discussed here, and also for a solvated globular protein,^19^ the pair correlation term Δ*S*_2_ dominates the solvent–solvent correlations Δ*S*_≥2_. Along the same lines, therefore, −*T*Δ*S*_≥2_ can be seen as the natural counterpart of Δ*U*_vv_, and in fact actually represents what the vv-subscript of −*T*Δ*S*_vv_, perhaps misleadingly, seems to suggest.

We hope our explanations and examples will contribute to resolving a long-standing controversy and the resulting widespread confusion. Fully in line with Ben-Naim’s theorem, solvent–solvent correlations can—and generally do—contribute markedly to the overall free energy of solvation, thus underscoring the need for an improved understanding of the “iceberg”-type ordering of solvent shells, in particular near complex macromolecular solutes and surfaces.

## References

[ref1] Israelachvili J. N.; Mitchell D. J.; Ninham B. W. J. Chem. Soc., Faraday Trans. 2 1976, 72, 1525–1568. 10.1039/f29767201525.

[ref2] De Vries A. H.; Mark A. E.; Marrink S. J. J. Am. Chem. Soc. 2004, 126, 4488–4489. 10.1021/ja0398417. 15070345

[ref3] Maibaum L.; Dinner A. R.; Chandler D. J. Phys. Chem. B 2004, 108, 6778–6781. 10.1021/jp037487t.

[ref4] Chandler D. Nature 2005, 437, 640–647. 10.1038/nature04162. 16193038

[ref5] Dias C. L.; Ala-Nissila T.; Wong-ekkabut J.; Vattulainen I.; Grant M.; Karttunen M. Cryobiology 2010, 60, 91–99. 10.1016/j.cryobiol.2009.07.005. 19616532

[ref6] Hummer G.; Garde S.; García A.; Pratt L. Chem. Phys. 2000, 258, 349–370. 10.1016/S0301-0104(00)00115-4.

[ref7] Frank H. S.; Evans M. W. J. Chem. Phys. 1945, 13, 507–532. 10.1063/1.1723985.

[ref8] Grabowska J.; Kuffel A.; Zielkiewicz J. J. Phys. Chem. B 2021, 125, 1611–1617. 10.1021/acs.jpcb.0c09489. 33539702 PMC7898264

[ref9] Head-Gordon T. Proc. Natl. Acad. Sci. U.S.A. 1995, 92, 8308–8312. 10.1073/pnas.92.18.8308. 11607575 PMC41146

[ref10] Noskov S. Y.; Lamoureux G.; Roux B. J. Phys. Chem. B 2005, 109, 6705–6713. 10.1021/jp045438q. 16851754

[ref11] Galamba N. J. Phys. Chem. B 2013, 117, 2153–2159. 10.1021/jp310649n. 23360515

[ref12] Ben-Naim A. Biopolymers 1975, 14, 1337–1355. 10.1002/bip.1975.360140704.

[ref13] Ben-Naim A.; Marcus Y. J. Chem. Phys. 1984, 81, 2016–2027. 10.1063/1.447824.

[ref14] Ben-Naim A. J. Chem. Phys. 2013, 139, 10B626_1 10.1063/1.4827086. 24182086

[ref15] Yu H.-A.; Karplus M. J. Chem. Phys. 1988, 89, 2366–2379. 10.1063/1.455080.

[ref16] Reynolds J. Nature’s Robots: A History of Proteins; Oxford University Press, 2001.

[ref17] Snyder P. W.; Lockett M. R.; Moustakas D. T.; Whitesides G. M. Eur. Phys. J.: Spec. Top. 2014, 223, 853–891. 10.1140/epjst/e2013-01818-y.

[ref18] Graziano G. J. Phys. Chem. B 2014, 118, 2598–2599. 10.1021/jp5008895. 24524630

[ref19] Heinz L. P.; Grubmüller H. Biophys. J. 2021, 120, 3470 10.1016/j.bpj.2021.05.019. 34087209 PMC8391029

[ref20] Heinz L. P.; Grubmüller H. J. Chem. Theory Comput. 2020, 16, 108–118. 10.1021/acs.jctc.9b00926. 31822062

[ref21] Heinz L. P.; Grubmüllerr H. J. Chem. Theory Comput. 2021, 17, 2090 10.1021/acs.jctc.0c00961. 33710881 PMC8047778

[ref22] Matsuda H. Phys. Rev. E: Stat. Phys., Plasmas, Fluids, Relat. Interdiscip. Top. 2000, 62, 3096–3102. 10.1103/PhysRevE.62.3096. 11088803

[ref23] Hnizdo V.; Darian E.; Fedorowicz A.; Demchuk E.; Li S.; Singh H. J. Comput. Chem. 2007, 28, 655–668. 10.1002/jcc.20589. 17195154

[ref24] Hnizdo V.; Tan J.; Killian B. J.; Gilson M. K. J. Comput. Chem. 2008, 29, 1605–1614. 10.1002/jcc.20919. 18293293 PMC2620139

[ref25] Fengler M. Estimating Orientational Water Entropy at Protein Interfaces. Ph.D. Thesis, Georg-August-Universität Göttingen, 2011.

[ref26] Persson R. A.; Pattni V.; Singh A.; Kast S. M.; Heyden M. J. Chem. Theory Comput. 2017, 13, 4467–4481. 10.1021/acs.jctc.7b00184. 28783431 PMC5607457

[ref27] Goethe M.; Fita I.; Rubi J. M. J. Chem. Phys. 2017, 147, 224102 10.1063/1.4996847. 29246041

[ref28] Berendsen H. J.; van der Spoel D.; van Drunen R. Comput. Phys. Commun. 1995, 91, 43–56. 10.1016/0010-4655(95)00042-E.

[ref29] Van Der Spoel D.; Lindahl E.; Hess B.; Groenhof G.; Mark A. E.; Berendsen H. J. J. Comput. Chem. 2005, 26, 1701–1718. 10.1002/jcc.20291. 16211538

[ref30] Hess B.; Kutzner C.; Van Der Spoel D.; Lindahl E. J. Chem. Theory Comput. 2008, 4, 435–447. 10.1021/ct700301q. 26620784

[ref31] Pronk S.; Páll S.; Schulz R.; Larsson P.; Bjelkmar P.; Apostolov R.; Shirts M. R.; Smith J. C.; Kasson P. M.; van der Spoel D.; Hess B.; Lindahl E. Bioinformatics 2013, 29, 845–854. 10.1093/bioinformatics/btt055. 23407358 PMC3605599

[ref32] Pall S.; Abraham M. J.; Kutzner C.; Hess B.; Lindahl E. Tackling Exascale Software Challenges in Molecular Dynamics Simulations with GROMACS; International Conference on Exascale Applications and Software, 2014; pp 3–27.

[ref33] MacKerell A. D. Jr.; Bashford D.; Bellott M.; Dunbrack R. L. Jr.; Evanseck J. D.; Field M. J.; Fischer S.; Gao J.; Guo H.; Ha S.; Joseph-McCarthy D.; Kuchnir L.; Kuczera K.; Lau F. T. K.; Mattos C.; Michnick S.; Ngo T.; Nguyen D. T.; Prodhom B.; Reiher W. E. III; Roux B.; Schlenkrich M.; Smith J. C.; Stote R.; Straub J.; Watanabe M.; Wiórkiewicz-Kuczera J.; Yin D.; Karplus M. J. Phys. Chem. B 1998, 102, 3586–3616. 10.1021/jp973084f. 24889800

[ref34] Huang J.; MacKerell A. D. J. Comput. Chem. 2013, 34, 2135–2145. 10.1002/jcc.23354. 23832629 PMC3800559

[ref35] Huang J.; Rauscher S.; Nawrocki G.; Ran T.; Feig M.; de Groot B. L.; Grubmüller H.; MacKerell A. D. Jr. Nat. Methods 2017, 14, 71–73. 10.1038/nmeth.4067. 27819658 PMC5199616

[ref36] Jones J. E. Proc. R. Soc. London, Ser. A 1924, 106, 463–477. 10.1098/rspa.1924.0082.

[ref37] Bussi G.; Donadio D.; Parrinello M. J. Chem. Phys. 2007, 126, 014101 10.1063/1.2408420. 17212484

[ref38] Berendsen H. J.; Postma J. P. M.; van Gunsteren W. F.; DiNola A.; Haak J. R. J. Chem. Phys. 1984, 81, 3684–3690. 10.1063/1.448118.

[ref39] Reinhard F.; Grubmüller H. J. Chem. Phys. 2007, 126, 014102 10.1063/1.2400220. 17212485

[ref40] Reinhard F.; Lange O. F.; Hub J. S.; Haas J.; Grubmüller H. Comput. Phys. Commun. 2009, 180, 455–458. 10.1016/j.cpc.2008.10.018.

